# The prevalence of feigning and concealment of Covid-19 infections in an international sample

**DOI:** 10.1177/13591053231226033

**Published:** 2024-01-28

**Authors:** Irena Boskovic, Esteban Puente-López, Brechje Dandachi-FitzGerald, Harald Merckelbach

**Affiliations:** 1 Erasmus University Rotterdam, The Netherlands; 2 Universidad de Valladolid, Spain; 3 Maastricht University, The Netherlands

**Keywords:** concealment, coronavirus, Covid-19, feigning, response bias, SARS-CoV2

## Abstract

We asked 463 participants from 21 countries whether they had feigned and/or concealed having a coronavirus infection during the pandemic period. 384 respondents (83%) reported having experienced a coronavirus infection. They were, on average, younger and reported more chronic health issues than participants who said they had never been infected. 65 (14%) admitted to having feigned the infection. Prevalence doubled (28%) when asked if they knew anyone who had feigned a coronavirus infection. Main motives for feigning were to stay at home and to obtain sick leave. As to having concealed a coronavirus infection, 56 (12%) responded affirmatively, but when asked about others, the prevalence reached 51% (*n* = 210). The most common reasons for concealment were to avoid letting others know and to not miss an event. Thus, both feigning and concealing infections can occur on a nontrivial scale, directly affecting prevalence rates in studies that rely on self-reported data collected from social platforms.

Until now, the severe acute respiratory syndrome covid-2 (SARS-CoV-2), or Covid-19 (i.e. coronavirus), has impacted more than 750 million people, causing more than 6 million deaths worldwide. The clinical picture of a coronavirus infection includes symptoms such as fever, cough, loss of taste, loss of smell, muscle aches and in some cases disorientation and vomiting (World Health Organization (WHO), 2023). Meta-analyses indicate that fever and cough are the most commonly reported symptoms, whereas other complaints vary in their frequency of occurrence (Grant et al., 2020; Li et al., 2020; see also Amin et al., 2021). Certain demographic factors, such as age, premorbid health and even gender, are known to modulate the course of Covid-19 infections (Gebhard et al., 2020; WHO, 2023). The overall prevalence of post-Covid-19 symptoms was estimated to be 50.6% in the communal samples and higher than 70% in the hospital setting (European Centre for Disease Prevention and Control, 2022).

Depending on the country, different regulations were implemented to lower exposure to the virus, such as working from home, mask-wearing, social distancing and quarantining when infected (WHO, 2020). People differed in their compliance with these measures, and their adherence was shown to be under the influence of culture, morality, pro-social tendencies and political orientation (Romano et al., 2021; Xu and Cheng, 2021). The restrictions often changed over time, introducing variability in adherence depending on, for instance, type of job and living situation. Thus, some people faced higher levels of job insecurity, were not given an option of working from home anymore (see Lin et al., 2021), and/or had to quarantine more frequently if living with others (see Bayer and Kuhn, 2020). Such challenges might have made some individuals more prone to distorting their health reports. For instance, making false claims of infection could justify working remotely, whereas suppressing information about an infection might allow bypassing quarantine and isolation measures.

When a person distorts their symptom report so that it no longer accurately reflects their health state, they exhibit a so-called *response bias*. Response bias in subjective health reports is a well-known phenomenon, and it typically takes either a negative (i.e. portraying your health as worse than it actually is) or a positive (i.e. overstating your health condition; Rogers, 2018) form. The prevalence of the negative response bias varies depending on many factors, such as the type of symptoms (physical or psychological, e.g. Boone et al., 2021, see also Greve at al., 2009; Mittenberg et al., 2002; Rogers, 2018), context of the symptom assessment (clinical or forensic; e.g. Martin and Schroeder, 2020), country (Nijdam-Jones and Rosenfeld, 2017) and, most importantly, the quality of gain a claimant wants to obtain (e.g. McDermott et al., 2013). When an external incentive is driving biased symptom reporting, this behaviour is also labelled as malingering and when incentives are not defined the preferred term is *feigning* (Rogers, 2018).

During the pre-covid period, Dandachi-FitzGerald et al. (2020) investigated the prevalence of feigning in a mixed Dutch sample using a proxy responders approach (i.e. ‘Do you know people who feign?’). More than half of responders (59%, *n* = 238; *N* = 401) said that they knew one or more persons who engaged or had engaged in feigning, with 34% (*n* = 137) having a direct confession from the feigner. When asked directly about their behaviour, a significantly lower, yet substantial number of respondents indicated that they themselves had feigned symptoms: 45% (*n* = 181). Headaches/migraines, cold/fever and stomachache were the most commonly fabricated complaints, used to obtain a sick leave, excuse a failure and/or seek attention from others (Dandachi-FitzGerald et al., 2020). This pattern was replicated in a Spanish sample (*N* = 1003), with 62.3% (*n* = 625) of the respondents knowing someone who feigned an illness, from which 57% (*n* = 357) had a direct confession from the feigner. Further, more than a third of the sample (34%, *n* = 341) admitted having feigned symptoms themselves. Headaches/migraines, neck pain and depression-anxiety related complaints were the most popular complaints used to secure a sick leave from work/school and monetary benefits (Puente-López et al., 2023). In both surveys, the preference for certain symptoms to be feigned was related to how easy it is to feign them and whether one has prior experience with these symptoms (i.e. familiarity; Dandachi-FitzGerald et al., 2020; Puente-López et al., 2023).

Considering the frequently cited symptoms of Covid-19 (such as fever and cough), and high familiarity of the public with the signs of the infection (see Anwar et al., 2020), clinical presentation of Covid-19 could be especially vulnerable to response bias. However, to the best of our knowledge, no study explored negative response bias (i.e. feigning) in Covid-19 reports. On the other hand, concealment of Covid-19 symptoms, that is, positive response bias or concealment has been studied, albeit on a small scale. Bearing in mind the stigma and public scrutiny of those who were not following the Covid-19 public health regulations (Bhanot et al., 2021; Rewerska-Juśko and Rejdak, 2022), it is not surprising that individuals were prone to align their health reports with socially favourable expectations. Also, given that hiding the infection had a greater negative impact on others’ health than feigning non-existing infections, it is understandable that symptom concealment received priority among researchers.

O’Connor and Evans (2022) explored dishonesty during the pandemic, and found that, among those who contracted Covid-19 (*n* = 86), a third (34%, *n* = 29) admitted denying having the infection, whereas more than a half (52%, *n* = 57) reported downplaying the symptoms to a certain degree. Men engaged in symptom concealment more frequently than women, and younger participants more often hid the infection than older subjects. Levy et al. (2022) confirmed that younger participants (<60 years) concealed Covid-19 infections more commonly and were more dishonest about their adherence to public health measures than older participants (>60). The age difference might be attributable to the heightened susceptibility of older individuals to complications from Covid-19. Older people may be more aware than their younger counterparts that misleading reports can lead to significant and widespread repercussions (O’Connor and Evans, 2022). However, a more thorough examination of the underlying reasons for concealment is still missing. Understanding the scale and drivers of response bias in symptom reporting, in either its negative or positive form, in the context of Covid-19 might help us prevent or counter such behaviour in any future public health threat.

## Current study

Considering the widespread occurrence and public knowledge of Covid-19,^1^ it is reasonable to speculate that certain individuals might have been inclined to pretend to have contracted Covid-19, or/and were tempted to conceal Covid-19 symptoms over the past 3 years. The former assumption has not yet been empirically tested, whereas the latter already gained some empirical evidence (O’Connor and Evans, 2022). Regardless of its direction (negative or positive), biased symptom reporting seriously impacts the accuracy of prevalence statistics (overestimated or underestimated) and, hence, it requires further research. Also, it is important to note that feigning and concealing are not mutually exclusive behaviours. Therefore, in this pioneering project, we investigated how often participants in an international sample had engaged in both negative (i.e. feigning) *and/or* positive (i.e. concealing) response bias in Covid-19 reports. We did so by asking participants to describe their own behaviour and behaviour of others around them during the last 3 years (March 2020–March 2023). As this project was exploratory in nature, no specific hypotheses were formulated.

## Method

### Participants

Initially, 646 people joined the study. However, their responses were screened using the following exclusion criteria: (1) informed consent not provided (*n* = 3), (2) permission to use their data not provided (*n* = 47), (3) not answering all the questions (*n* = 127), (4) under legal age (<18, *n* = 3) and (5) proper elaboration on the study at the end (*n* = 3, see Procedure). The final sample consisted of 463 participants, with an average age of 29 years (SD = 12.33; range 18–84). With 70.8% (*n* = 328) females were overrepresented; 28.3% (*n* = 131) identified as men, and four participants identified as non-binary. Because health regulations differed across countries, we asked participants where they spent most of the time during the pandemic period (previous 3 years). The following countries were mentioned: the Netherlands (48.1%), Greece (32.2%), Croatia (6.6%) and USA (4.2%), countries where they were also currently residing.

We collected other demographic background information about our sample, such as education, employment, income and living situation (see Table 1). Most participants had either a high school qualification (48.6%) or a bachelor’s degree (30.2%). Regarding employment status, the most frequently endorsed categories were full time employment (35.4%) and being a student (38%). Almost half earned a yearly income between 10,000 and 20,000 euros (45.5%), and almost half of the sample lived with their parents (45.6%). As living situation could have an impact on one’s willingness to report Covid-19 infection, we checked whether participants lived with a person who would be, under the WHO advice, considered vulnerable to the virus (i.e. people older than 60 and/or with pre-existing respiratory or immune system related issues^2^). A third of the sample, indeed, lived with a vulnerable person (36.4%).

**Table 1.** Demographic details of the sample (*N* = 463).

	Percentage	Frequency
Education (obtained diploma)		
Elementary school	2.8	13
High school	48.6	225
Bachelor	30.2	140
Master’s	16	74
PhD	2.4	11
Employment status		
Full time employed	35.4	164
Part-time employed	17.3	80
Student	38	176
Yearly income		
10k–20k	45.3	210
20k–30k	21.4	99
Living situation		
With parents	45.6	211
With partner	16.8	78
With flatmates	11.7	54
Other (mixed options)	13.8	64
Living with a vulnerable person		
Yes	36.5	160
Not sure	2.8	13
No	54.4	252

Finally, participants rated their current health on 5-point measure (lower values indicating poorer health), and whether they had any chronic health complaints (yes/no). The average health rating was ‘good’ (*M* = 3.95, SD = 0.76, range 1–5), and 17.7% (*n* = 82) indicated having chronic health issues, such as asthma, allergies, migraines and blood pressure problems.

### Questionnaire

We created a questionnaire for the purpose of this study containing 36 questions (see Supplemental file). The first part contained demographic questions (cf. supra Table 1). Next, a question about having experienced a coronavirus infection was presented, and when participants indicated that they had had such infection in the past, they were queried about the frequency of infections and the type of symptoms they experienced. People who claimed to not have had a coronavirus infection would not be presented those questions; accordingly, the number of questions presented varied across participants depending on their answers. Next, we provided a question about whether they had feigned a coronavirus infection during the period of 3 years, which was the time interval between the start of the pandemic and this study. Depending on their answer, participants were given different follow up questions. For instance, if feigning was indicated, then this was followed-up by questions about the type of the symptoms a person had been feigning and the motivation for doing so. If a participant reported not having feigned a coronavirus infection, they were asked about others and whether they knew anyone who had been feigning a coronavirus infection. The same procedure was followed for questions regarding coronavirus symptoms concealment. This structure of protocol was employed to secure that the study duration and task load were relatively similar for all participants. All participants were asked to rate how frequently the general public engages in feigning (i.e. pretending to have) and concealing (i.e. pretending *not* to have) Covid-19 infections using a 5-point Likert scale (1 being never and 5 indicating all the time).

### Procedure

We used Qualtrics to administer questions to participants. We, an international group of researchers, employed convenience sampling by distributing an online questionnaire to people in our professional and private networks, as well as posting the study on social media (Twitter, Facebook). In the invitation letter, we provided basic information about the study and informed participants that their participation was purely voluntary, that data would be processed in an anonymized way, and that they would be joining a lottery for a €10 voucher. After agreeing to participate, participants needed to provide informed consent and only then they were presented the questionnaire (see above). Our inquiry about participants’ symptom (mis)reporting specified the time period of 3 years prior to their participation, which took place during March, April and May 2023. After answering all of the questions, participants were asked to briefly describe the study (i.e. elaboration of the task), in order for us to ensure that participants were attentively responding. Thus, this question served as an elimination criterion (*n* = 3). Lastly, participants had to evaluate their participation (motivation, clarity, honesty), and were given a debriefing form, after which they were asked if they explicitly provide a permission to use and store their data according to the ethical requirements at our institution. The study was approved by the standing Ethical Committee of Erasmus University Rotterdam, the Netherlands.

### Data analyses

The data were first screened using the exclusion criteria (see Participants section). After exclusion of non-eligible participants, descriptive analyses were run. To inspect group differences, we used Welch *t-*test and Mann Whitney *U*-test (see Delacre et al., 2017). In doing so, we applied Bonferroni corrections on the basis of the number of comparisons. The data file and the outputs are available at Open Science Framework (https://osf.io/tv4k3/?view_only=8f5b2cfbdd4e4f8fb7d83faa63bef8b7).

## Results

### Participation evaluation

Participants had to rate their motivation, the clarity of questions, potential discomfort they had experienced and their honesty using 5-point scales (with lower values indicating lower levels and higher values higher levels). Participants indicated moderate motivation (*M* = 3.35, SD = 0.91, range 1–5), high clarity of questions (*M* = 4.43, SD = 0.71, range 2–5), low discomfort (*M* = 1.35, SD = 0.90, range 1–5) and high honesty (*M* = 4.88, SD = 0.35, range 3–5).

### Covid-19 infection prevalence

Three hundred eighty-four participants (82.9%) indicated having had a Covid-19 infection in the past. Most participants had the infection once or twice, but some indicated to have suffered from it five times (maximum value). The most prevalent symptoms were exhaustion, blocked nose, fever and cough (see Table 2).

**Table 2.** Prevalence of coronavirus symptoms for self-declared authentic and feigned instances.

	Authentic infections		Feigned infections		χ^2^(1)	*p*
*N*	384		65			
Symptoms	*n*	%	*n*	%		
Cough	277	72.1	44	67.7	1.08	0.30
Fever	283	73.7	41	63	3.16	0.075
Blocked nose	284	74	23	35.4	**38.24**	**<0.001**
Sore throat	267	69.5	22	33.8	**30.81**	**<0.001**
Sneezing	210	54.6	13	20	**26.56**	**<0.001**
Muscle/body aches	264	68.7	22	33.8	**29.2**	**<0.001**
Loos of taste	128	34	9	13.8	**10.6**	**0.002**
Loss of smell	134	35	7	10.8	**15.05**	**<0.001**
Brain fog	127	33	5	7.7	**17.12**	**<0.001**
Exhaustion	306	79.7	28	43.1	**39.01**	**<0.001**
Other (chest pain, dizziness, headaches)	61	16	3	4.6	5.86	0.015

Note: *p* level was adjusted to <0.005. The results presented in bold were statistically significant.

To test for age differences between those who reported having experienced a Covid-19 episode (*n* = 384) and those who said they did not (*n* = 79), we performed a Welch *t*-test. The first group was, on average, younger (*M* = 27.95, SD = 11.04) than the second group (*M* = 33.78, SD = 16.53), Welch *t*(92.81) = 3.00, *p* = 0.003, Cohen’s *d* = 0.48. Further, those who reported to have had the infection, more often reported chronic health issues compared to those who did not report having attracted the coronavirus: Mann Whitney U = 13,545, *z* = 2.26, *p* = 0.023, *r* = 0.11. There were no differences between these groups in terms of gender, education, employment or income.

### Prevalence of feigned Covid-19 infections

Sixty-five participants (14.1%; 95% CI [11, 17]) admitted having feigned a Covid-infection at least once. Of these, 55 (9%) said they completely fabricated it, 1.1% (*n* = 5) reported feigning previously existing symptoms even after the infection had passed and 3.5% (*n* = 16) had a normal cold but pretended it was a Covid-19 infection. Participants, on average, engaged in feigning twice, with maximum reported frequency of 10 instances. The most commonly reported symptoms in feigned cases were cough, fever and exhaustion (see Table 2). Compared with self-declared authentic infection reports, feigned instances included less frequently all other typical symptoms, such as blocked nose, exhaustion, loss of smell and taste and muscle aches. The majority of those who had engaged in feigning a Covid-infection were familiar with the condition as they also reported being infected in the past (81.5%, *n* = 53).

Main reasons for feigning were preferring to stay at home (52%, *n* = 34), to obtain sick leave from school or work (38.5%, *n* = 25), to avoid commitments (35.4%, *n* = 23) and to avoid risky situations to protect others (24.6%, *n* = 16). Two participants also indicated academic benefits (e.g. deadline extensions) and four reported excusing a failed exam by referring to an infection.

Using Mann Whitney *U*-tests, we checked whether there were differences between participants who said they had feigned (*n* = 65) and those who said they had not (*n* = 398) in terms of age, gender, education, health ratings and living situation (i.e. whether they lived with a vulnerable person). There were no such differences, all Mann Whitney Us < 12,147.5, *Z_s_* < 1.56, *p_s_* > 0.128.

### Participants’ estimation of feigning prevalence

Those who said they had never feigned a coronavirus infection (*N* = 398) were asked whether they knew others who had feigned it; 32.7% (95% CI [28, 37]; *n* = 130) said they knew such a person. A vast majority got the confirmation directly from the feigner (82.3%, *n* = 107), whereas others were informed by third parties (7.7%, *n* = 10), and still others said their intuition told them (4.6%, *n* = 6). When asked which symptoms the feigners falsely claimed, participants most frequently indicated being told about cough (64%, *n* = 84) and fever (65.4%, *n* = 85), whereas blocked nose (37.7%, *n* = 49) and exhaustion (31.5%, *n* = 41) were less often mentioned, a pattern that corresponds well with the prevalences in Table 2. We also asked whether they knew about the motivation for feigners, and the most often mentioned motives were to stay at home (61%, *n* = 79), obtain sick leave (67.7%, *n* = 88) or avoid duties/commitments (31.5%, *n* = 41), which again reflects the outcomes of feigners’ self-report presented above.

All participants were asked to rate, using a 5-point scale (range from *never* to *all the time*), how often general public engages in pretending to have coronavirus infection. Only 7.1% (*n* = 33) said never, whereas the rest of the sample (92.9%, *n* = 430) indicated different frequencies, with the majority indicating from time to time (55%, *n* = 236), whereas a considerable minority said rarely (33.5%, *n* = 144). Forty-seven participants (11%) reported that others feigned the condition frequently (*n* = 47), and three participants said all the time.

### Prevalence of concealed Covid-19 infections

Fifty participants (11.8%, 95% CI [6, 12]) said they had previously experienced a Covid-19 infection but had kept this hidden. On average, two instances of concealment were reported (range 1–3). Twenty-one (42%) knew they had the infection but claimed to only have a normal cold, 15 (30%) falsely claimed that their infection had passed although they still had it, and 14 (28%) claimed to be completely healthy even though they knew they were infected.

Main self-declared motives were not wanting others to know and make it a ‘big deal’ (32%, *n* = 16), not wanting to miss out on a party/festival (38%, *n* = 19), and not wanting more days off (20%, *n* = 10). The rest of the responses indicated having other obligations (e.g. exams, important meeting), and avoiding responsibility for spreading the virus to friends.

Participants who concealed a coronavirus infection (*n* = 50) differed only in one aspect from those who said they had never engaged in concealment (*n* = 413): the first group was younger than the second, Mann Whitney *U*_s_ = 7815.5, *Z* = 2.79, *p* = 0.005, *r* = 0.11. There were no significant group differences for gender, education, health ratings or living situation.

### Participants’ estimation of denial prevalence

The rest of the sample (*n* = 413) answered the question whether they were aware of anyone who had concealed having a coronavirus infection despite actually having contracted it. Two-hundred-ten participants (50.8%, 95% CI [46, 56]) indicated they did. The majority said to have had a direct confession from the concealer (55.2%, *n* = 116), 20.0% (*n* = 42) stated that the symptoms were obvious so there was no need for confirmation, and 14.3% (*n* = 30) got the evidence from other sources (e.g. mutual friends). Interestingly, 7.1% (*n* = 15) mentioned other methods of confirmation – such as having witnessed the positive test, or knowing that the concealer did not ‘believe in corona’ so that they would not test, nor admit it.

The motives participants assumed to play a role in denying having a coronavirus infection despite being ill were not wanting to miss out on a party/festival (29%, *n* = 134), not wanting others to know and make a big issue out of it (18%, *n* = 83), not wanting to stay at home (13%, *n* = 60) and not wanting more days off (8%, *n* = 37). Around 30 participants added responses that alluded to deniers being ‘anti-vaxxers’, who did not want to acknowledge that the virus is real.

All participants were again asked to provide their estimations, using a 5-point scale, about how often people engage in hiding coronavirus infection. Only 3% (*n* = 14) indicated that people never do that, whereas 97% reported different degrees of frequency. The majority (57%, *n* = 263) reported that people concealed Covid-19 moderately often, 20.5% (*n* = 95) said that people do it most of the time and 18% (*n* = 83) indicated that others hide coronavirus infection rarely, whereas eight participants thought it happens always.

### Feigning and denial

Out of the entire sample (*N* = 463), only seven participants (1.5%) acknowledged that they had participated in both fabricating a Covid-19 infection in the past and, on other occasions, denying such an infection even though they were aware of having contracted the virus. Hence, it seems that people rather opt for one form of response bias.

## Discussion

This study was the first attempt to explore the occurrence of both feigning (i.e. negative response bias) and concealment (i.e. positive response bias) in Covid-19 reports. These two forms of response bias may potentially obscure the precision of Covid-19 prevalence estimates and may also have had adverse consequences for the safety of individuals and the economy.

The main results can be summarized as follows: First, the majority of our sample (>80%) had contracted Covid-19 in the past, on average 1–2 times, most frequently experiencing symptoms such as exhaustion, blocked nose, fever and cough. People who reported having had coronavirus in the past were on average younger than those who did not. Given that older population was particularly at risk for medical complications, it is possible that older participants in our survey took more preventive measures and were more cautious than younger participants. Previous research found, indeed, that younger participants had lower adherence to the health restrictions (e.g. keeping social distance; Levy et al., 2022), and were more often infected by coronavirus (Doerre and Doblhammer, 2022). Further, those who have had experienced Covid-19 reported more chronic health issues than participants who were not infected. The connection between episodes of Covid-19 infection and chronic health issues has been noted by other researchers as well (Del Rio et al., 2020) and it makes sense: having had the infection in the past may have cascaded chronic health problems.

Second, 14% of our sample admitted having feigned a coronavirus infection in the past, with the majority completely fabricating the infection and the rest exaggerating a common cold. As to feigned Covid-19 infection symptoms, their profile was less rich than that of self-declared authentic episodes although for both authentic and feigned episodes fever and cough were the most frequently endorsed symptoms. The difference in symptom profiles might have to do with the successive Covid-19 variants that emerged. Fever and cough were consistently reported as the most common signs of coronavirus (Grant et al., 2020), whereas symptoms such as loss of smell and/or taste seem to be more specific signs of certain (temporal) virus variants (Whitaker et al., 2022). Further, fabrication of, for instance, loss of smell or taste would require more effort and time from feigners than short-term symptoms such as fever and cough. Relatedly, the vast majority of those who feigned symptoms of coronavirus also reported having the infection in the past, which corresponds with previous findings about feigners opting for symptoms they are familiar with (Dandachi-FitzGerald et al., 2020; Puente-López et al., 2023).

The main motivation to falsely report having Covid-19 was to stay at home or get sick leave. These incentives and motives resonate with previous research on feigning (Dandachi-FitzGerald et al., 2020; Puente-López et al., 2023). There were no demographic differences between those who did and those who did not engage in feigning coronavirus infection, confirming that feigning is not a behaviour exhibited in some specific population, but rather a part of a common behaviour spectrum (Dandachi-FitzGerald et al., 2020).

Interestingly, the prevalence of feigning Covid-19 (14%) was noticeably lower than prevalence numbers reported in prior studies looking at feigning in the general public (30%–50%; Dandachi-FitzGerald et al., 2020; Puente-López et al., 2023). Although there are similarities in the type of the symptoms we tested, it is important to note that such symptoms (e.g. headaches, cold) in two previous studies were not framed as a sign of an *infectious* disease like in our survey. Thus, the discrepancy can be explained by two scenarios: (a) that people engage in feigning significantly less often when it comes to coronavirus because of its global impact and/or because Covid-19 is easy to detect using the (highly sensitive) tests, or (b) that people are considerably less open to disclose feigning of Covid-19 infections specifically due to public scrutiny.

Third, and related to the previous point, when participants were asked to report about *other peoples’ behaviour*, the prevalence of feigning Covid-19 infection doubled, with 28% of our sample stating that they knew someone who had engaged in this type of behaviour. This number is much closer to the estimated prevalence reported in previous research on feigning (34%, Dandachi-FitzGerald et al., 2020). The majority of our participants had direct confirmation from the person, and they were accurate in addressing the others’ motivation for feigning, with staying at home and sick leave being the main drivers of such behaviour.

Fourth, a non-trivial minority (12%) admitted falsely denying (i.e. concealing) a coronavirus infection. From these, the majority minimised the health complaints (i.e. stating that it is a cold), and a smaller fraction fully denied the infection. The main motives behind concealment were either not to have others know about the infection or not to miss out on being with others. In the former case, it is possible that people who concealed coronavirus infection wanted to avoid social stigma (Bhanot et al., 2021; Rewerska-Juśko and Rejdak, 2022), whereas the latter motive illustrates why concealment is a riskier type of behaviour than feigning in the context of an infectious disease. Not surprisingly, as fear of missing out (i.e. ‘fomo’) is often found to be typical for younger populations (Hayran and Anik, 2021), we found that younger participants engaged more frequently in concealment than older ones. This aligns with our finding that younger people more often reported episodes of Covid-19 infections than older people, and it is in line with the extant literature showing that younger adults more often engage in deceptive behaviour than older people (O’Connor et al., 2022).

The proportion of participants who knew someone who falsely denied having coronavirus infection was 51%. This prevalence is higher than when participants were asked about knowing someone who feigned the infection, which aligns with the idea that in many domains, hiding symptoms occurs on a larger scale than symptom fabrication (Rogers, 2018). Further, the prevalence reported in a previous study on concealment of Covid-19 (52%, with varying degrees of concealment, O’Connor and Evans, 2022) was very similar to the one we found. In our study, the majority of participants who knew about concealment of a Covid-19 infection had a direct confirmation from the person, whereas the rest stated that others’ symptoms were too obvious, confirming our prior consideration that hiding an infection is more challenging than feigning one. A good portion of participants explained that the others who concealed being sick did so because they were against the vaccines and did not believe that the virus actually exists. This is a recurrent issue in the broader literature on faking good: that people who have skeptical attitudes towards (psychological or physical) illness or towards seeking help might be more prone to concealment (Rogers, 2018). Indeed, previous research indicated that people with doubts about the coronavirus exhibited lower adherence to the health measures than those who were not distrustful (Lee et al., 2020).

Overall, when asked about their estimation of both feigning and concealment of Covid-19 in the general public, 93% and 97% of our participants, respectively, indicated that such behaviours occur on a varying degree. Looking at the overlap between the two forms of symptom mispresentation, only 1.5% of our participants engaged in both feigning and concealing Covid-19 symptoms at some point. Hence, it appears that people mostly opted for one specific form of response bias.

### Limitations and future directions

Our findings need to be considered in the context of this survey’s limitations. Although our sample was international, the vast majority of our participants were Dutch, Greek or American. Future surveys should try to expand to other countries, especially those that were known for having different (if any) restrictions during the pandemic, such as Sweden, which did not have any restrictions. It would be important to investigate whether a lack of such regulations prevented or significantly lowered response bias in self-declared Covid-19 episodes, especially concealment. Further, the majority of our sample were students under the age of 30, meaning that the generalizability of our findings is limited. Considering the findings about younger participants exhibiting more concealment regarding Covid-19 infections (O’Connor and Evans, 2022), better representation of different age groups seems like a necessary next step. More importantly, in order to ensure that the survey length was similar to all of our participants, some participants were not shown the same questions as others. For instance, those who directly admitted engaging in feigning were further questioned about symptoms, motivations etc., whereas those who denied feigning were asked about knowing others who fake. This way, we did not have a chance to hear from ‘feigners’ themselves whether they knew others who behaved in a similar way. Although all participants were later asked to rate the prevalence of faking in the general public, this oversight should be corrected in further investigations. Moreover, we did not directly ask about the impact the pandemic had on our participants, for instance, on their job security or living situation, which were both shown to be related to people’s way of reporting Covid-19 infections (Bayer and Kuhn, 2020; Lin et al., 2021). Finally, as our data suggest that the attitude towards coronavirus was one of the main motives for others to deny having the infection, we realized too late that asking participants about their views on coronavirus and vaccinations would have been an informative question that could potentially be related to the prevalence in which they feigned or concealed Covid-19. Thus, the issue of personal perception of the pandemic and its impact on one’s behaviour should be further explored in the follow-up research.

A more fundamental issue is, of course, whether people in a survey would admit feigning or denying a Covid-19 infection; and whether those who admit such behaviour can be trusted in all the other information that they provided. Given these concerns, we believe that the safest conclusion that can be drawn from our data is that feigning and concealment of Covid-19 infections did occur on a non-trivial scale.

## Conclusion

Overall, our investigation was a first attempt to document how often people misreported their Covid-19 status. The results provide room for optimism. For one, a minority of 14% admitted feigning a coronavirus infection, while 12% said that had falsely denied it (i.e. concealed it). Although these prevalences are non-trivial, they are also considerably lower than the estimated prevalence of feigning in the prior literature, which could be explained by our focus on an infectious disease with a direct impact on others. Admittedly, when we asked participants about faking behaviour in those around them, the prevalence numbers increased to 28% and 51%, respectively, suggesting that symptom concealment was more prevalent than feigning. This discrepancy is worrisome, especially in the context of an infectious disease. Drawing on the Health Belief Model (HBM; Rosenstock, 1974) as a framework for understanding health-promoting behaviours, this study suggests a nuanced view of individuals’ actions during the pandemic. The HBM is based on the premise that personal beliefs about health problems, perceived benefits of action and barriers to action can explain engagement (or lack of engagement) in health-promoting behaviour. Some participants may view feigning a coronavirus infection as a socially responsible choice, albeit with some economic consequences, while preventing risk to others, aligning with the HBM’s perceived benefits. On the other hand, concealing an infection, despite its public health risks, indicates a potential underestimation of the disease’s severity and personal susceptibility, as postulated by the HBM. This suggests that immediate personal gains, like attending events, are prioritized over collective health safety. However, these percentages do certainly not imply that 28% and 51% of the social networks of our respondents engaged in feigning or denial. It may well be that our respondents had similar cases in mind and that these cases reflect only a small proportion of the entire social network.

Nevertheless, our research underscores the importance of approaching studies relying on self-reported data about Covid-19 infections (such as Ames et al., 2021; Wu et al., 2020) with a degree of caution. Our findings suggest that the prevalence estimates of Covid-19 obtained through self-report studies may lack accuracy. It’s crucial to note that the presence of response bias is not exclusive to Covid-19 research but may be inherent in any data dependent on self-reports. Consequently, our study could serve as an encouragement for fellow researchers to incorporate validity checks, such as Symptom Validity Tests, in their investigations. By integrating response bias assessments into their surveys, health researchers have the potential to enhance the reliability and validity of their conclusions.

## Supplemental Material

sj-docx-1-hpq-10.1177_13591053231226033 – Supplemental material for The prevalence of feigning and concealment of Covid-19 infections in an international sample Supplemental material, sj-docx-1-hpq-10.1177_13591053231226033 for The prevalence of feigning and concealment of Covid-19 infections in an international sample by Irena Boskovic, Esteban Puente-López, Brechje Dandachi-FitzGerald and Harald Merckelbach in Journal of Health Psychology

## Notes

1. In this manuscript, the terms Covid-19 and coronavirus are used interchangeably.

2. See https://www.who.int/westernpacific/emergencies/covid-19/information/high-risk-groups
