# Supplementary material for: The prevalence of feigning and concealment of Covid-19 infections in an international sample
Source: J Health Psychol. 2024 Jan 28;29(6):595–607. doi: 10.1177/13591053231226033 (PMC11075400; doi:10.1177/13591053231226033)
Supplement: sj-docx-1-hpq-10.1177_13591053231226033 – Supplemental material for The prevalence of feigning and concealment of Covid-19 infections in an international sample [file sj-docx-1-hpq-10.1177_13591053231226033.docx]

# SUPPLEMENTAL FILE

**The Prevalence of Feigning and Concealment of Covid-19 Infections in an International Sample**

# Invitation Letter Your Corona Experience

Hereby, we invite you to join a brief survey about your corona experience so far. We have only a few questions for you, after which you will receive the debriefing form in which we explain the purpose of this survey. Your participation will not take longer than 10-15 minutes, and it will be pseudonymised, meaning that you will be asked to provide a unique code to which you can refer in case you do not want your data to be included.

Your participation is voluntary and in case you are a student of [blinded for review], you will be rewarded with research credits, otherwise, you will be asked to join a lottery for a €10 voucher. There are no foreseen risks in participating in this survey. For any questions or remarks, please contact the principal researcher, [blinded for review].

Best,

The research team

Dear participant,

# Informed consent Your Corona Experience

Thank you for your interest in this survey about your Corona experience. We will first ask you some demographic questions (e.g., age, gender, country etc.), after which we will present you questions about the pandemic, and finally, we will have some queries about your view on our survey.

Your participation is voluntary and will not take longer than 15 minutes. You will be asked to provide a unique code in case you think you will want to withdraw your data. Students of Erasmus University will be asked to leave their student IDs so that we can assign research credits for you. Others will be asked to enter their email addresses to join the lottery for a

€10 voucher. This information will not be downloaded nor linked to your data and will be destroyed once the data collection is completed.

The purpose of this project is to inspect a scientific research question and use the collected data for scientific purposes (writing a manuscript and presenting the data at conferences). The data will be stored at [blinded for review] document vault (for a minimum of 10 years) and on the Open Science Framework. The access to the data will have to be approved by the principal researcher of the project[blinded for review].

There are no foreseen risks of participating, and you can stop your participation at any point just by existing the link without providing any explanation and without consequences. For any questions and remarks, please contact the principal researcher [blinded for review].

By responding below you indicate that a) you fully understood above provided information,

1. that you are above 18 years of age, and c) that you consent to participation in this project:

Yes No

# Corona Experience Questionnaire

- 1. **Demographic questions:**
     - Age
     - Gender (optional)
     - Education level
     - Current place of residence
     - How would you rate your current health on a 5-point scale (poor to excellent)
     - Do you have any chronic issues: Yes (optional to elaborate), No

# Corona-related questions

1. **Have you been infected by the Corona virus**: Yes – please indicate a) how many times b) the time of each infection; No; Maybe (I had some symptoms but I did not test)
   1. For those who responded “Yes” and “Maybe” – Below is the list of symptoms typical for Corona infection (please indicate the presence and/or intensity of each):
      1. Cough –
      2. Fever –
      3. Blocked nose –
      4. Sore throat –
      5. Sneezing –
      6. Muscle/body aces –
      7. Loss of taste –
      8. Loss of smell –
      9. Brain fog –
      10. Exhaustion –
      11. Other symptoms to add: (open form)
   2. For those who responded “No” – Which of the following symptoms do you think are the most commonly present and intense in a Corona infection:
      1. Cough –
      2. Fever –
      3. Blocked nose –
      4. Sore throat –
      5. Sneezing –
      6. Muscle/body aces –
      7. Loss of taste –
      8. Loss of smell –
      9. Brain fog –
      10. Exhaustion –
      11. Other symptoms to add: (open form)
2. (for all participants**) Have you ever, during this past two and a half years, claimed to have Corona knowing that you did not have Corona**: Yes, I said I had it although I didn’t at the time; Yes, I said I had it for a longer time that it was the case (my actual infection already passed); Yes, I had a normal cold, but I reported that it was Corona; No.
   1. (for those “yes”) – **How many times have you intentionally misreported Corona infection:**
   2. (for those “yes”) – **please indicate which of the symptoms did you feign to have (in all occasions)**
      1. Cough
      2. Fever
      3. Blocked nose
      4. Sore throat
      5. Sneezing
      6. Muscle/body aces
      7. Loss of taste
      8. Loss of smell
      9. Brain fog
      10. Exhaustion
      11. Other symptoms to add:
   3. (for those yes) **What was your motivation to do so**:
      1. Getting attention/Help from others
      2. I wanted to stay at home and do work/school online
      3. To excuse a failure
      4. To obtain medication
      5. Sick leave from work/school
      6. More liberal academic regulations (e.g., extra resit)
      7. To avoid social commitments I did not want to go to (e.g., party, festival, date etc.)
      8. Something else:

# For those No: Do you know anyone who claimed to have Corona although they knew they did not have Corona at the time: Yes, No

- - 1. If Yes: How do you know they were feigning:

1. They told me so
2. I felt it/My intuition told me
3. Others told me
4. Their symptoms were not credible
5. Something else:

# What kind of symptoms did they claim:

1. Cough
2. Fever
3. Blocked nose
4. Sore throat
5. Sneezing
6. Muscle/body aces
7. Loss of taste
8. Loss of smell
9. Brain fog
10. Exhaustion
11. Other symptoms to add:

# What do you think was their motivation to feign having the Corona infection:

1. Attention/Help from others
2. I wanted to stay at home and do work/school online
3. To excuse a failure
4. To obtain medication
5. Sick leave from work/school
6. More liberal academic regulations (e.g., extra resit)
7. To avoid social commitments I did not want to go to (e.g., party, festival, date etc.)
8. Something else:

# How often, in general, do you think others have feigned having Corona infection during the pandemic?

- 1. Never
  2. Rarely
  3. From time to time
  4. Frequently
  5. All the time

1. (for all participants) **Have you ever, during this past two and a half years, denied having Corona although you knew you had it at the time**: Yes, I said I do not have it although I did at the time; Yes, I said I do not have anymore, although that was not the case (my actual infection was still ongoing); Yes, I had Corona, but I reported that it was just a normal cold; No.

# (for those “yes”) – How many times have you claimed not to have Corona infection although you did at the time:

- 1. **What was the motivation behind this decision**:
     1. I did not want attention/help from others
     2. Because others would make it a big deal out of it and I did not want others to know
     3. I did not want to miss out on something (e.g., party, festival etc.)
     4. I did not want to stay at home and do my work/school online
     5. I did not want more days off from work/school
     6. I was lonely and wanted company
     7. I did not want to lose an appointment (e.g., dentist, vaccination, etc.)
     8. Something else:

# For “No” - Do you know anyone who denied having Corona although they knew they did have Corona at the time: Yes, No

- - 1. If Yes: How do you know they were lying:
       1. They told me so
       2. I felt it (my intuition told me)
       3. Others told me
       4. Their symptoms were obvious
       5. Something else:

# What do you think was their motivation to deny having the Corona infection:

- - - 1. They did not want attention/help from others
      2. Because others would make it a big deal out of it and they did not want others to know
      3. They did not want to miss out on something (e.g., party, festival etc.)
      4. They did not want to stay at home and do my work/school online
      5. They did not want more days off from work/school
      6. They were lonely and wanted company
      7. They did not want to lose an appointment (e.g., dentist, vaccination, etc.)
      8. Something else:

# How often, in general, do you think others have falsely denied having Corona during the pandemic?

- 1. Never
  2. Rarely
  3. From time to time
  4. Frequently
  5. All the time

# Exit Questions: Before we end the survey, we have a couple of questions regarding your participation.

- 1. How interested/motivated were you to join this survey? 5-point scale (not at all- extremely)
  2. How clear were the questions we asked? 5-point (not at all – extremely)
  3. Could you, in a couple of words, please explain what this survey was about? This is to ensure that you properly understood our questions and that you were paying attention (open space)
  4. How uncomfortable were the questions we asked to you? 5-point (not at all –

extremely)

- 1. Do you have any comments or remarks you wish to share with us? (open form)

# Debriefing form

Dear participant,

Thank you for responding to our questions. As you have noticed, this survey was about your Corona experience. Namely, we have been in a pandemic for longer than two years now, and Corona has become a part of our lives, and if not you, many around you have certainly experienced the infection by the coronavirus. It was established that many people have different experiences of Corona symptoms and have shown different severity of the infection. Yet, people also differ in the way they report symptoms. For instance, in the field of symptom validity assessment (forensic psychology), it is estimated that approximately 30% of the general public feigns everyday, common symptoms (Dandachi-Fitzgerald et al.,

2020). Also, we know that a certain proportion of the population also hides symptoms, although we do not have a close estimation of how frequent that behaviour is. The most frequently reported reasons for such behaviour are to obtain something (like compensation) or to justify a failure. Yet, the reasons behind peoples’ decision to deceive about their health are highly related to situational factors and difficult to predict. Hence, in this survey, we wanted to investigate how often have people feigned or hidden the Corona infection during the previous two and a half years. Further, we wanted to establish the most commonly feigned symptoms and the most impactful reasons for health-deceptive behaviour. We do not have any clear predictions as this survey is exploratory.

If you wish to be informed about our findings, please leave your email address below.

1. **I want to be informed about the findings of this survey** (this information will not be linked to your data) – open form for the email address
2. **In case you think you would want to withdraw your data, please leave a code to which you can refer** (we suggest using your mother’s initials and your date of birth – e.g., LO02051989)

# Do we have Your permission to store your (pseudonymised) data? Yes - No

1. **Are you a student of** [blinded for review]:
   1. **Yes** - please leave your student ID so that you can receive your research credits – open form (THIS INFORMATION WILL NOT BE USED FOR ANY OTHER PURPOSES AND WILL NOT BE STORED)
   2. **No** - if you wish to join the lottery for a €10 voucher, please leave your email address

For any questions or remarks, please contact [blinded for review]

Thank you for your time.
